# In vivo imaging of *Lactococcus lactis*, *Lactobacillus plantarum* and *Escherichia coli* expressing infrared fluorescent protein in mice

**DOI:** 10.1186/s12934-015-0376-4

**Published:** 2015-11-14

**Authors:** Aleš Berlec, Janja Završnik, Miha Butinar, Boris Turk, Borut Štrukelj

**Affiliations:** Department of Biotechnology, Jožef Stefan Institute, Jamova 39, 1000 Ljubljana, Slovenia; Department of Biochemistry and Molecular and Structural Biology, Jožef Stefan Institute, Jamova 39, 1000 Ljubljana, Slovenia; Centre of Excellence for Integrated Approaches in Chemistry and Biology of Proteins, Jamova 39, 1000 Ljubljana, Slovenia; Faculty of Chemistry and Chemical Technology, University of Ljubljana, 1000 Ljubljana, Slovenia; Faculty of Pharmacy, University of Ljubljana, Aškerčeva 7, 1000 Ljubljana, Slovenia

**Keywords:** In vivo imaging, Lactic acid bacteria, *Lactococcus lactis*, *Lactobacillus plantarum*, *Escherichia coli*, Infrared fluorescent protein, Mice

## Abstract

**Background:**

In vivo imaging of orally administered lactic acid bacteria (LAB) and commensal bacteria in mice is shown to provide information on the spatial and temporal distribution of bacteria in the gastrointestinal tract. The bacteria can be detected and monitored using bioluminescence or near-infrared fluorescence.

**Results:**

Fluorescence imaging of bacteria was established by expressing the infrared fluorescent protein IRFP713 in *Lactococcus lactis*, *Lactobacillus plantarum* and *Escherichia coli.* All three bacterial species were monitored in live mice and no major differences in transit time were observed. Bacteria passed through the stomach and small intestine in 1 h and the majority were secreted from the large intestine after 6–8 h. Intestinal localization of bacteria was confirmed by imaging the isolated intestines and culturing the intestinal content. The use of fluorescence tomography for spatial localization of fluorescent bacteria has been established. The expression of an additional infrared fluorescent protein IRFP682 enabled concomitant detection of two bacterial populations in live mice.

**Conclusions:**

The present work provides a methodological basis for future studies of probiotic and theranostic actions of LAB in mouse disease models.

**Electronic supplementary material:**

The online version of this article (doi:10.1186/s12934-015-0376-4) contains supplementary material, which is available to authorized users.

## Background

In vivo optical imaging is a non-invasive method for spatial and temporal monitoring of bacteria in live animals. It can provide data on bacterial dissemination in real time and enables better use of lower numbers of experimental animals. In vivo imaging is of particular importance in the study of infectious diseases [1] and has been used to monitor the progression of infection with *Salmonella typhimurium*, *Pseudomonas aeruginosa*, *Streptococcus pneumoniae* and *Listeria monocytogenes*, etc. [1]. The majority of bacterial infection studies have been performed using bioluminescence, requiring the expression of luciferase, an enzyme that is often of bacterial origin (e.g. from *Vibrio* spp.) [1]. An alternative to bioluminescence is fluorescence. The latter requires an external light source, involves lower sensitivity and lower signal-to-noise ratio due to tissue autofluorescence. On the other hand, the fluorescence also has several advantages in comparison to bioluminescence: it does not require the administration of luciferin (which is time consuming and expensive), yields brighter signal and therefore requires shorter exposure times, and is more appropriate for combining with single-cell in vitro assays such as microscopy or flow cytometry [2–4]. Tissue autofluorescence is minimal in the near infrared region between 700 and 1000 nm [5]. Although this spectral range is covered by organic fluorescent probes [6], they require an appropriate labelling technique and are less suitable for bacteria since they are diluted with cell division. Infrared fluorescent proteins (IFP1.4 [7] and IRFP [8]) with absorption and emission maxima in the near infrared region have recently been developed and expressed constitutively in bacteria. IFP1.4 has been obtained by mutagenesis of bacteriophytochrome DrBphD from *Deinococcus radiodurans* [7], and IRFP by mutagenesis of RpBphP2 from *Rhodopseudomonas palustris* [8]. Both fluorescent proteins require heme catabolic product biliverdin as a covalently-bound exogenous chromophore [8]. IRFP (aka IRFP713; GenBank accession number AEL88490) has excitation/emission maxima at 690/713 nm. Additional mutagenesis of IRFP yielded new variants with slightly different spectral properties, including IRFP682 (GenBank accession number AGN32864) with excitation/emission maxima at 663/682 nm [9]. Spectra of two proteins overlap; however they can be distinguished in vivo by spectral unmixing [9].

Lactic acid bacteria (LAB) are used routinely in the food industry and have an excellent safety record. LAB, particularly *Lactobacillus* spp. and *Bifidobacterium* spp., are also gaining importance in therapy as probiotics, due to their beneficial health effects [10–12]. Their intrinsic probiotic activity can be improved by the use of genetic engineering [13]. Genetically modified probiotic LAB can serve as vectors for local delivery of biologically active molecules to the gastrointestinal tract or other mucosal surfaces, facilitating rational targeting of pathology-related molecules [14–17]. The ability to track probiotic, as well as commensal bacteria such as *E. coli*, in the digestive tract of live animals would provide valuable insights of probiotic action and their interaction with commensal bacteria. Daniel et al. [18] have expressed luciferases of different origins in *Lactobacillus plantarum* and *Lactococcus lactis* in order to study bacterial persistence and precise localization in the intestine. They have shown luciferase from beetle *Pyrophorus plagiophthalamus* to be the most appropriate for in vivo imaging.

Several fluorescent proteins, including GFP and mCherry, have been expressed in LAB and used for the study of intestinal tract colonization in mouse, chicken and zebrafish [19–23]. However, due to shorter excitation and emission wavelengths of the fluorophores that overlap with high haemoglobin absorption and strong tissue autofluorescence, the animals had to be euthanized and their organs examined ex vivo.

We report the expression of IRFP in prototype LAB *L. lactis* and *Lb. plantarum*, and in prototype commensal/pathogen bacterium *E. coli* to monitor and compare the bacteria in vivo in mice by the use of fluorescence. Different imaging modalities that are supported by IVIS Spectrum in vivo imager were tested. Expression of IRFP713 and IRFP682 allowed simultaneous detection of two different bacteria in a single animal by using the spectral unmixing algorithm.

The current work aims at establishing an effective in vivo fluorescence imaging platform for beneficial bacteria. In vivo imaging is expected to be one of the crucial research tools in future probiotic studies, by enabling spatiotemporal monitoring of probiotic bacteria, their interaction with the immune system and with both commensal and pathogenic bacteria. The ability to concurrently track different bacterial species will be of the greatest importance for the latter.

## Results

### Construction of infrared fluorescent *L. lactis*, *Lb. plantarum* and *E. coli*

**Fig. 1:**
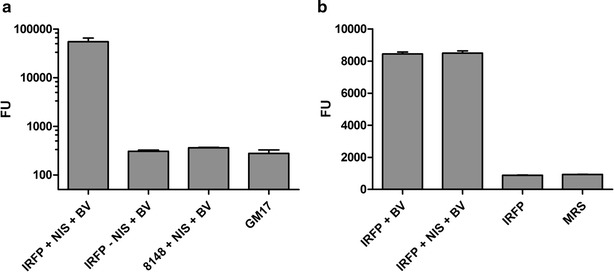
Fluorescence intensity of *L. lactis* (**a**) or *Lb. plantarum* (**b**) expressing IRFP713 in comparison to various controls. IRFP denotes pNZ-IRFP713 (**a**) or pNZRK-IRFP713 plasmid (**b**). Biliverdin (BV) or nisin (NIS) were added where noted (+). *8148* pNZ8148 empty vehicle control (without *irfp713* gene), *GM17* M17 medium supplemented with glucose, *MRS* De Man, Rogosa and Sharpe medium

**Fig. 2:**
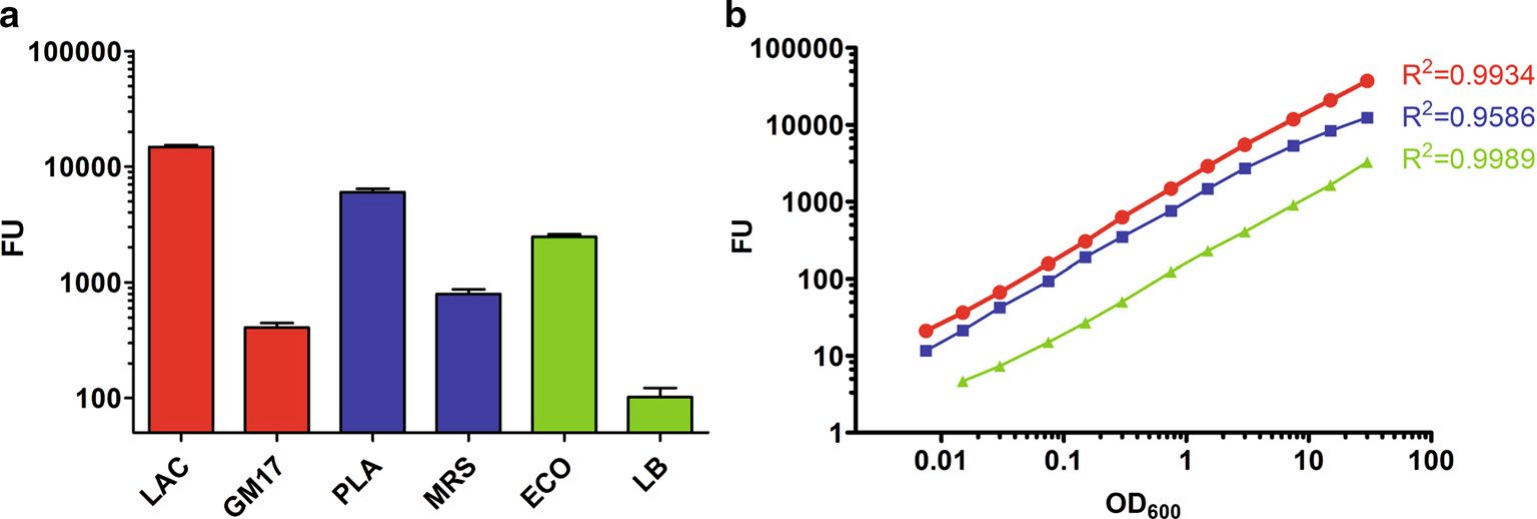
Fluorescence intensity of *L. lactis* (LAC; *red*), *Lb. plantarum* (PLA; *blue*) and *E. coli* (ECO; *green*) expressing IRFP713. **a** Fluorescence intensity was normalized to OD_600_ = 1.0. Growth media of corresponding bacteria (*GM17* M17 medium with 0.5 % glucose, *LB* lysogeny broth, *MRS* De Man, Rogosa and Sharpe medium) were used as negative controls. **b** Fluorescence intensity of bacterial cells as a function of optical density (cell concentration). Correlation coefficients (R^2^) for individual *curves* are depicted in corresponding *colors*. *FU* fluorescence units, *OD*_*600*_ optical density

*Irfp713* open reading frame was cloned into different expression vectors for the expression of IRFP713 in different hosts*. Irfp713* was expressed under the control of PnisA promoter from pNZ-IRFP713 plasmid in *L. lactis* NZ9000 that contains genomic copies of *nisRK* genes and enables induction with nisin [24]. The fluorescence intensity of IRFP713-expressing *L. lactis* was much higher than that of the background fluorescence of uninduced culture, vehicle control culture (containing plasmid without *irfp713* ), or growth medium; the fluorescence intensities of the latter three were almost the same (Fig. 1a). Initial drop in fluorescence intensity from 43,000 to 25,000 FU was noted during 2 days of storage. However, after the initial drop of the fluorescence of the *L. lactis* culture, the latter remained relatively stable over the course of 42 days at 4 °C. High bacterial viability was enumerated during the first 14 days of storage (Additional file 1: Figure S1). In comparison to *L. lactis*, constitutive expression of *irfp713* (loss of responsiveness to nisin induction; Fig. 1b) was noted in *Lb. plantarum*, which was attributed to the introduction of *nisRK* genes into the backbone of pNZ-IRFP713 [25, 26]. In *E. coli*, *irfp713* was expressed under the control of constitutive CP25 promoter in pGEM::CP25-IRFP713 plasmid [27]. IRFP713 was successfully produced in all three expression hosts, albeit different normalized fluorescence intensities to the cell concentration were observed (Fig. 2a). The highest normalized fluorescence intensity was detected in *L. lactis*, followed by *Lb. plantarum* and *E. coli*, respectively (Fig. 2a). Fluorescence of the IRFP713-expressing *L. lactis*, *Lb. plantarum* and *E. coli* cultures increased linearly with increasing numbers of cells (Fig. 2b). Significant differences in fluorescence were observed over a broad bacterial concentration range (OD_600_ = 0.075–30.0), yielding a linear line on a log–log graph (Fig. 2b) with correlation coefficients exceeding 0.95 for *Lb. plantarum* and 0.99 for *L. lactis* and *E. coli*, respectively.

**Fig. 3:**
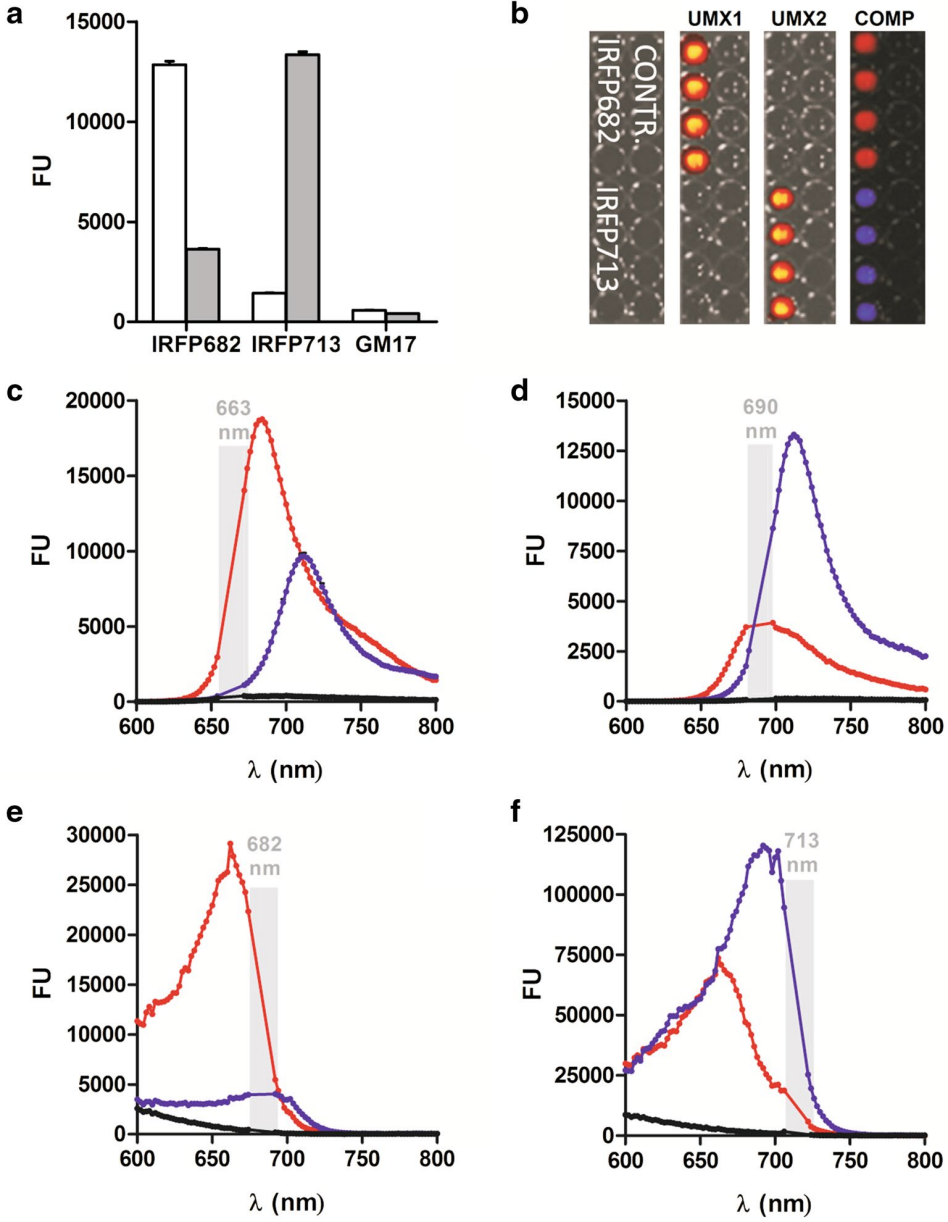
Evaluation of the expression of IRFP682 and IRFP713 in *L. lactis.* **a** Fluorescence intensity of *L. lactis* expressing IRFP682, IRFP713 and growth medium (GM17) determined at excitation/emission wavelengths 663/682 nm (*white*) or 690/713 nm (*gray*). **b** Distinction of IRFP682 and IRFP713-expressing lactococci, dispensed in microtiter plates, with IVIS Spectrum and spectral unmixing (*Contr.* empty vehicle control, *UMX1* IRFP682 fluorescence, *UMX2* IRF713 fluorescence, *COMP.* composite image). Emission (**c**, **d**) and excitation (**e**, **f**) spectra of IRFP682-expressing *L. lactis* (*red*), IRFP713-expressing *L. lactis* (*blue*) and control *L. lactis* (*black*), recorded at wavelengths specified in *gray*. *Gray* belts denote excitation and emission wavelengths at which reliable read-outs could not be obtained due to the vicinity of the wavelength used to record the spectrum

*Irfp682* gene was prepared by site-directed mutagenesis of the *irfp713* gene [27] and expressed in *L. lactis* NZ9000, as reported for the *irfp713* gene. Fluorescence intensities of the bacterial cultures expressing IRFP682 and IRFP713 were recorded at emission/excitation wavelengths 663/682 nm (optimal for IRFP682) and 690/713 nm (optimal for IRFP713; Fig. 3a). As expected, IRFP682-expressing bacteria yielded stronger fluorescence intensity at 663/682 nm and IRFP713-expressing bacteria at 690/713 nm. The absolute fluorescence intensities of the two IRFP-expressing bacterial populations at their respective optimal excitation and emission wavelengths were similar. Spectral overlap was observed as reported [9], fluorescence of both proteins being observed at both emission/excitation wavelengths. The fluorescence intensity of IRFP682-expressing bacteria at 690/713 nm was significantly higher than that of the control; however, it was threefold lower than that at 663/682 nm. Similarly, the fluorescence intensity of IRFP713-expressing bacteria at 663/682 nm was significantly higher than that of the control but lower than that of the IRFP682-expressing bacteria at 690/713 nm (Fig. 3a). The signals of IRFP682 and IRFP713-expressing bacteria dispensed in a microtiter plate could be completely separated with the IVIS Spectrum in vivo imager by guided spectral unmixing (Fig. 3b). The spectral properties of IRFP682- and IRFP713-expressing bacteria have been determined (Fig. 3c–f) and had excitation and emission maxima in accordance with those reported [9], which confirms the identity of the respective proteins. The control bacteria yielded almost no fluorescence and no excitation or emission maxima were observed (Fig. 3c–f).

### In vivo reflectance (epifluorescence) time-course imaging of mice following oral administration of IRFP713-expressing bacteria

**Fig. 4:**
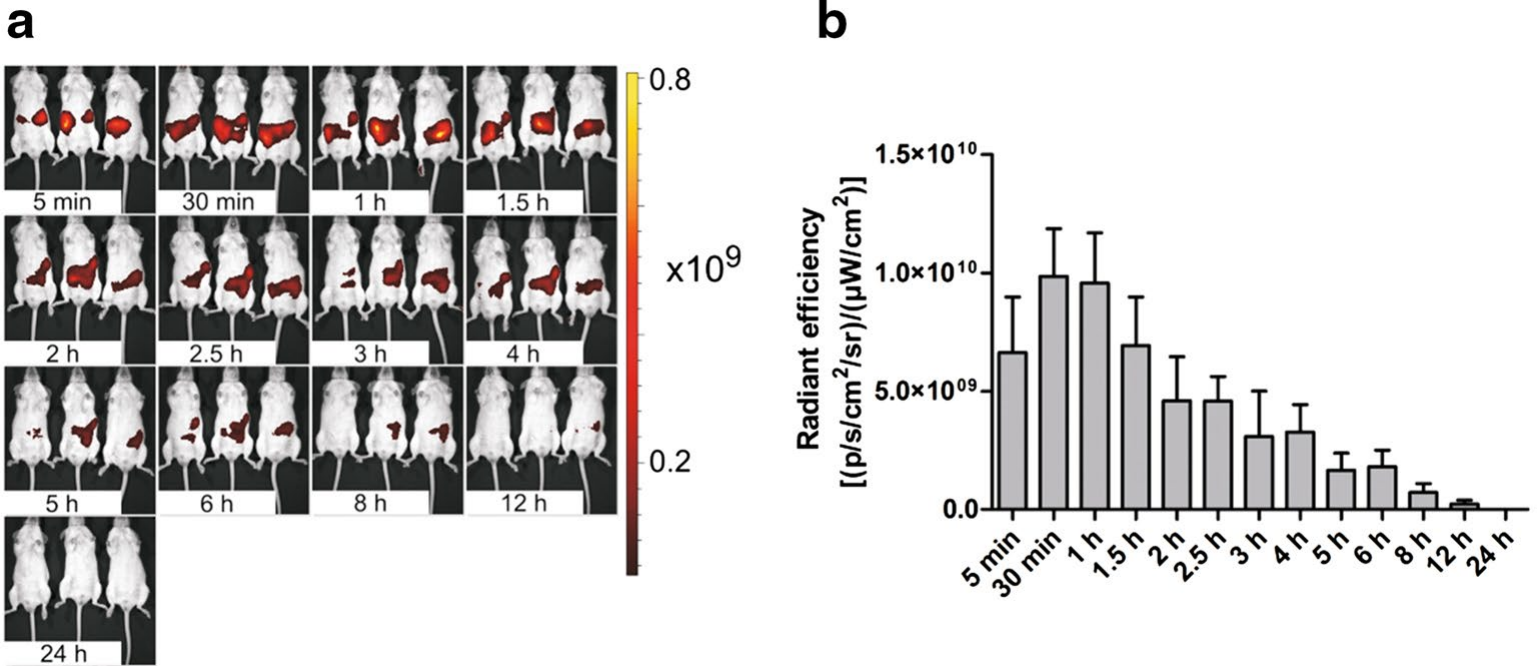
Time-course (24 h) imaging of mice administered with 5.0 × 10^10^ cells of IRFP713-expressing *L. lactis*. **a** Reflectance (epifluorescence) image with a *color bar* indicating radiant efficiency [(p/s/cm^2^/sr)/(μW/cm^2^)]. **b** Radiant efficiency of IRFP713-expressing *L. lactis* in mice as a function of time. *Vertical bars* indicate standard error

Three mice were administered 5.0 × 10^10^ IRFP713-expressing *L. lactis* cells to determine the time profile of the IRFP713 signal following oral administration of bacteria (Fig. 4). The IRFP713 signal at a given time point was reconstituted from a sequence of nine images recorded with different filter pair combinations using spectral unmixing (Additional file 2: Figure S2). Localization of the bacterial mass changed over time and was similar in all three mice (Fig. 4a). Radiant efficiency achieved maximum level (9.86 × 10^9^–9.56 × 10^9^) between 30 min and 1 h after the administration. It then decreased gradually over the course of 24 h, indicating secretion and bacterial dilution along the entire volume of the intestinal tract. After 8 h, only weak radiant efficiency was observed in two mice (2.27 × 10^8^), and after 12 h in only one mouse. After 24 h no fluorescence was observed, indicating elimination of the *L. lactis* bacteria from the intestine (Fig. 4).

**Fig. 5:**
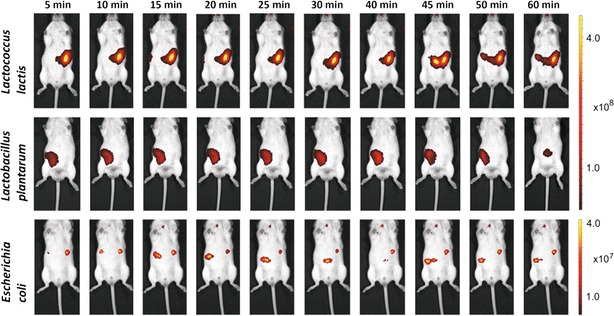
Representative time-course (1 h) imaging of mice receiving 5.0 × 10^10^ cells of IRFP713-expressing *L. lactis* (*upper row*), *Lb. plantarum* (*middle row*) and *E. coli* (*bottom row*). *Color bars* indicate radiant efficiency [(p/s/cm^2^/sr)/(μW/cm^2^)]

To visualize the dynamics of the bacterial mass during the first hour after administration, a time course was recorded at 5–10 min intervals following administration of 5.0 × 10^10^ cells of IRFP713-expressing *L. lactis*, *E. coli* and *Lb. plantarum* (Fig. 5). All three bacterial species were readily detected in mice although the average radiant efficiency was different between the species (1.3 × 10^8^*E. coli*, 1.9 × 10^9^*Lb. plantarum*, 4.6 × 10^9^*L. lactis*), due to the differences in normalized fluorescence observed between the different bacterial cells (Fig. 1). The exception was *E. coli* that yielded a fivefold lower signal than expected. The fluorescence intensity for a particular species remained relatively constant during the 1 h time course; although differences in localization of bacterial mass were readily observed.

### Ex vivo epifluorescence time-course imaging of mouse intestine following oral administration of IRFP713-expressing bacteria

**Fig. 6:**
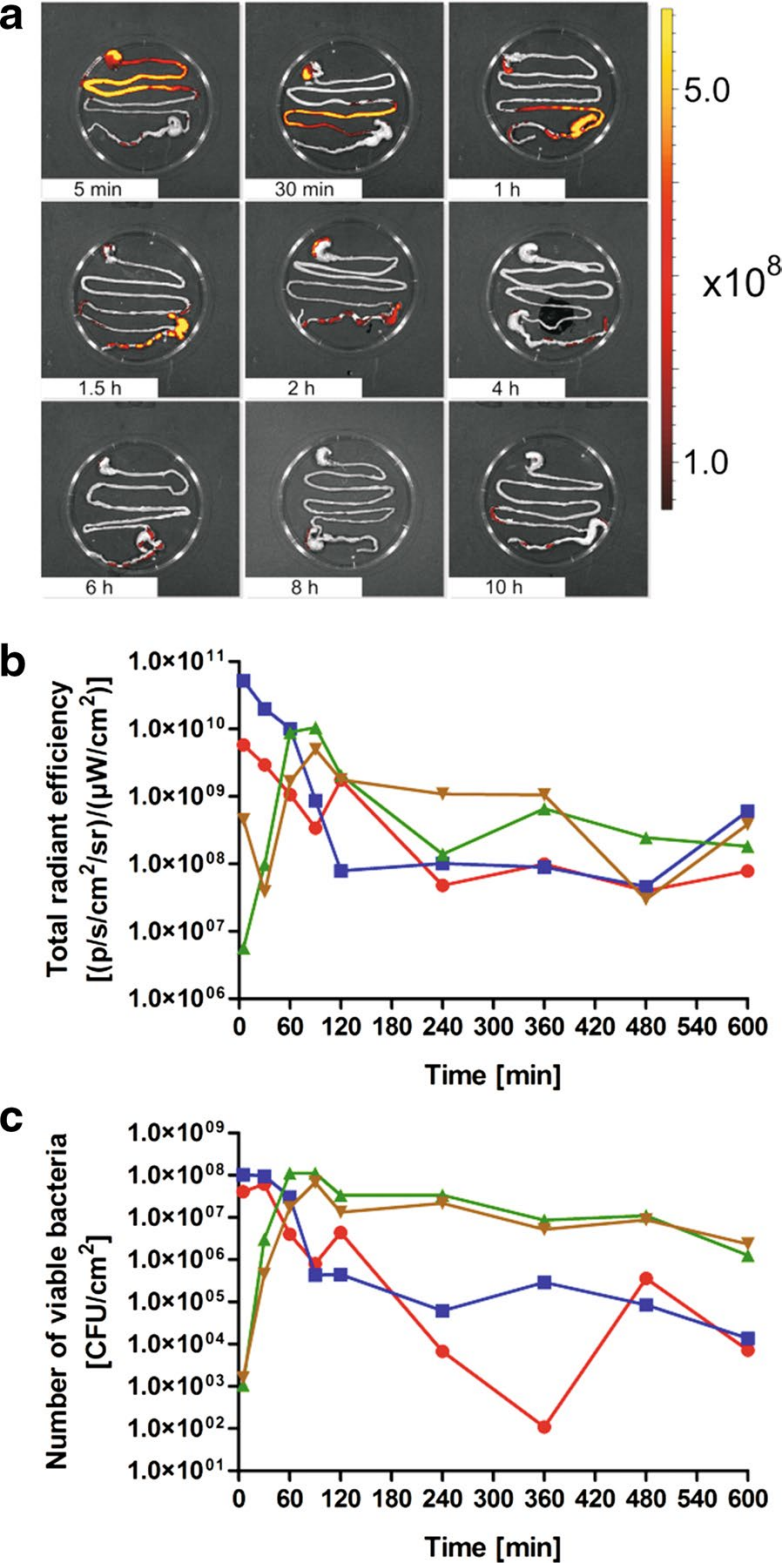
Transit and survival of IRFP713-expressing *L. lactis* in mice. Mice were administered with 5.0 × 10^10^ cells. Intestines were extracted at different time points and recorded by epifluorescence imaging (**a**). Radiant efficiencies [(p/sec/cm^2^/sr)/(μW/cm^2^); **b**] and numbers of viable bacteria (CFU/cm^2^; **c**) were determined in different parts of the intestine as a function of time. Stomach is shown in *red*, small intestine in *blue*, caecum in *green*, and large intestine in *brown*

The passage of 5.0 × 10^10^ cells of IRFP713-expressing *L. lactis* through individual parts of the intestine (stomach, small intestine, caecum, large intestine) as a function of time was determined by removing their intestines at specified time points and epifluorescence imaging (Fig. 6a). Bacteria were quantified by determining radiant efficiencies in individual parts of the intestine (Fig. 6b). Radiant efficiencies correlated with the number of viable bacteria (CFU/cm^2^) isolated from different parts of the intestine (Fig. 6c) at corresponding time points. The strongest correlation was calculated for caecum (R^2^ = 0.970) and large intestine (R^2^ = 0.951), but lower for stomach (R^2^ = 0.622) and small intestine (R^2^ = 0.819). Bacterial boluses were detected in the stomach and in the small intestine 5 min after administration. In the first 60 min bacteria passed through the small intestine and reached the caecum. They were retained in the caecum for several hours; from there they gradually cleared to the large intestine from which more than 90 % were secreted in 10 h, as observed by the decrease in both, CFU number and radiant efficiency (Fig. 6b, c).

**Fig. 7:**
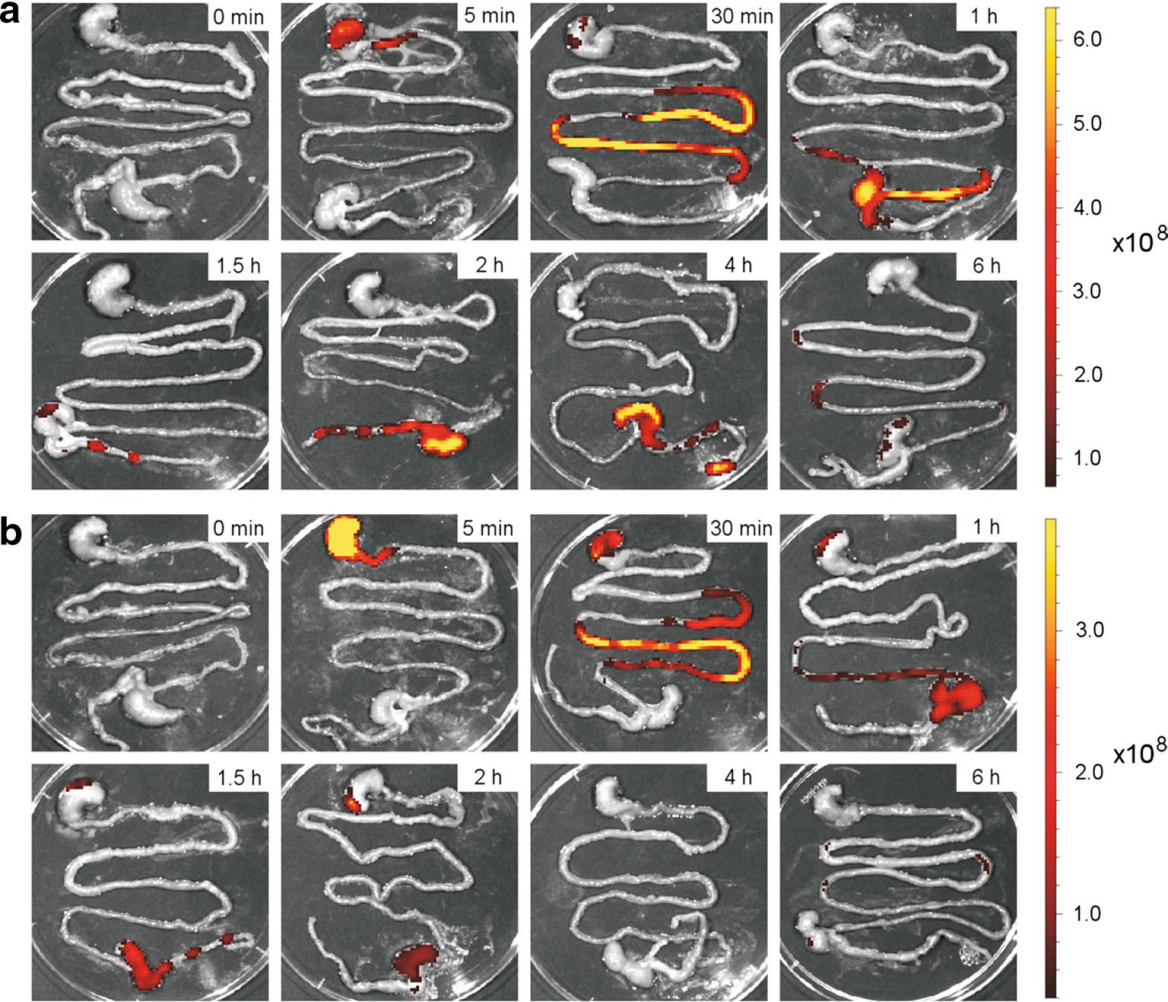
Ex vivo imaging of mouse intestines extracted at different time points after administration of 5.0 × 10^10^ cells of IRFP713-expressing *Lb. plantarum* (**a**) and *E. coli* (**b**). *Color bars* indicate radiant efficiency [(p/s/cm^2^/sr)/(μW/cm^2^)]

Mice were also administered with 5.0 × 10^10^ cells of IRFP713-expressing *E. coli* and *Lb. plantarum*. Similar profiles of intestinal transit as with *L. lactis* were observed in isolated intestine for both *Lb. plantarum* (Fig. 7a) and *E. coli* (Fig. 7b). However, the *E. coli* signal diminished 4 h after administration, indicating faster secretion or lower intensity of the *E. coli* signal, which was further weakened by bacterial dilution.

### Time-course fluorescence tomography imaging of IRFP713-expressing bacteria

**Fig. 8:**
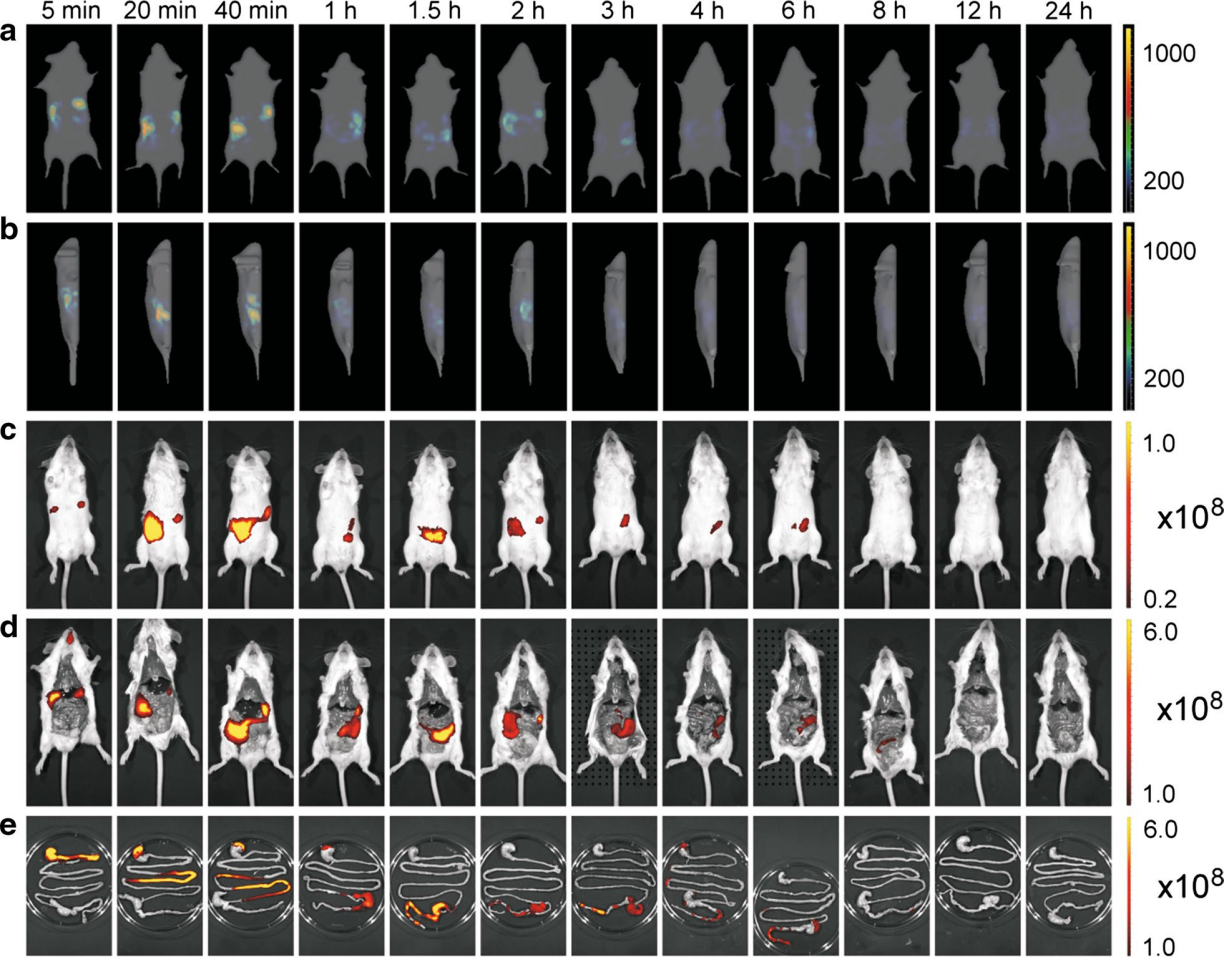
Imaging of mice at different time points after administration with 5.0 × 10^10^ cells of IRFP713-expressing *L. lactis*. Whole-body imaging was performed using trans-illumination and fluorescence imaging tomography (FLIT; **a** on the back, **b** on the side); or **c** epifluorescence. Ex vivo epifluorescence imaging was performed on dissected animals (**d**) or isolated intestines (**e**). *Color bars* indicate radiant efficiency [(p/s/cm^2^/sr)/(μW/cm^2^); epifluorescence] or total fluorescence yield (pmol/M cm; FLIT)

Eleven mice were administered with 5.0 × 10^10^ cells of IRFP713-expressing *L. lactis* and euthanized at defined time points over the course of 24 h. Fluorescence imaging tomography (FLIT) images of intact mice were recorded at each time point, using trans-illumination, and compared with the epifluorescence images of intact mice, images of mice with open abdominal cavity, and with the epifluorescence images of isolated intestines. Very good correlation of spatial distribution of bacterial mass was observed in the first 3 h after administration between FLIT images, epifluorescence images and epifluorescence images of mice with open abdominal cavity (Fig. 8). Precise localization of bacterial mass could be determined using isolated intestine. Clear spatial separation between stomach (5 min), small intestine (20, 40 min), caecum (60 min) and large intestine (1.5, 2 h) was observed. The radiant efficiencies of the intestines decreased over time due to the considerable secretion, from 4.37 × 10^9^ to 5.85 × 10^9^ during the first hour, to 1.13 × 10^8^–2.84 × 10^8^ in the period of 3–6 h after administration, and 5.54 × 10^6^–9.24 × 10^6^ in the period of 8–12 h after administration.

### Differentiation between IRFP682 and IRFP713-expressing bacteria in vivo and in isolated intestine

**Fig. 9:**
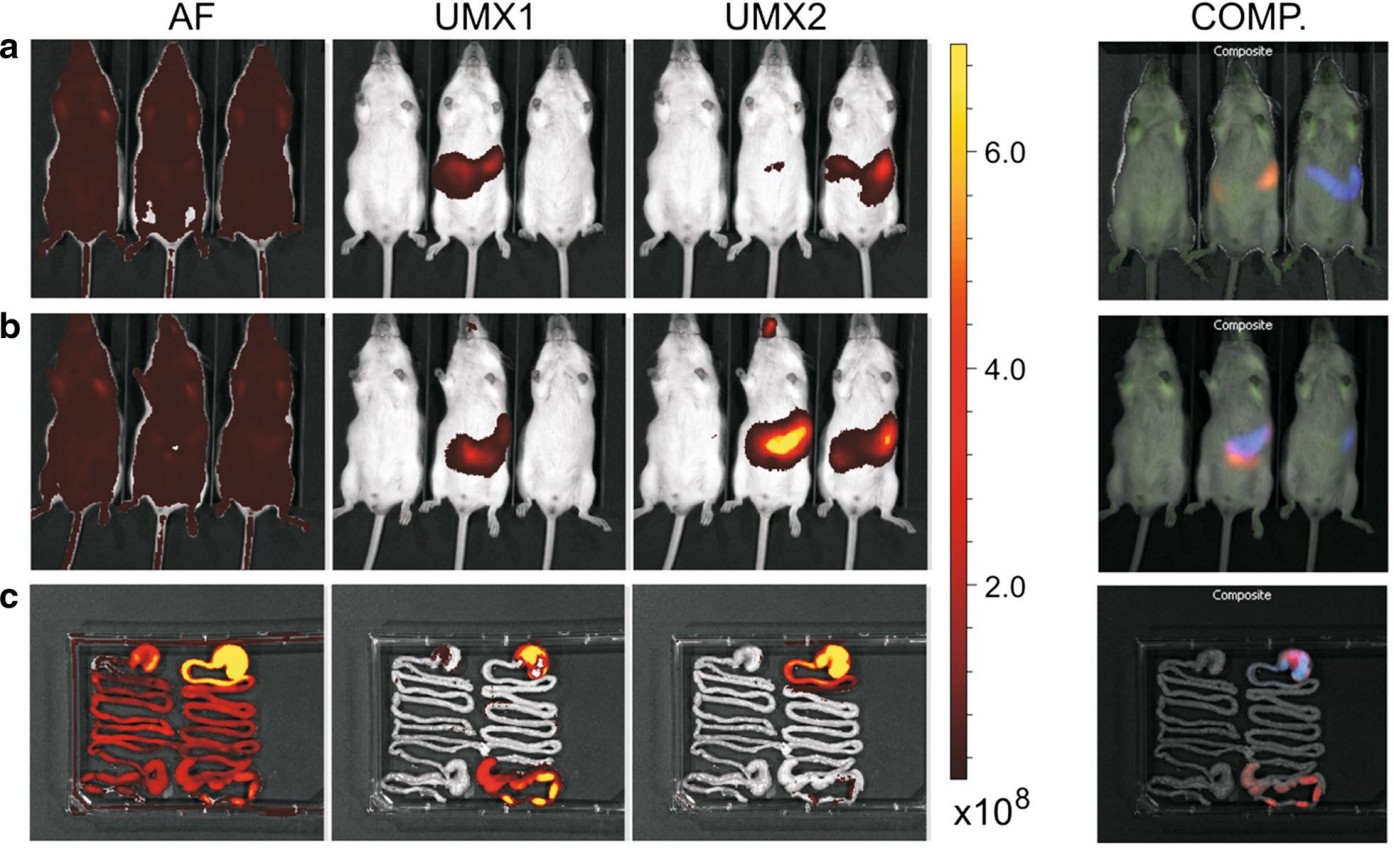
Concomitant imaging of two bacterial populations. **a** In vivo imaging 5 min after administration of 5.0 × 10^10^ cells of IRFP682-expressing *L. lactis* (*middle* mouse) and 5.0 × 10^10^ cells of IRFP713-expressing *L. lactis* (*right* mouse). Control mouse (*left*) received no bacteria. **b** In vivo imaging of mice 2 h after administration. The *middle* mouse was administered with an additional dose of 5.0 × 10^10^ cells of IRFP713-expressing *L. lactis* immediately prior to the imaging. **c** Ex vivo image of mouse intestine from a separate experiment. *Left* control mouse. *Right* mouse administered with IRFP682- and, after 2 h, IRFP-713-expressing *L. lactis*. *AF* tissue autofluorescence, *UMX1* IRFP682 fluorescence, *UMX2* IRFP713 fluorescence, *COMP.* composite image. *Color bar* indicates radiant efficiency [(p/sec/cm^2^/sr)/(μW/cm^2^)]

To verify the differentiation between IRFP682 and IRFP713-expressing bacteria in vivo, mice were administered with 5.0 × 10^10^ cells of IRFP682- and IRFP713-expressing *L. lactis*. Spectral unmixing was used to distinguish the fluorescence of IRFP682, IRFP713 and background tissue autofluorescence (Additional file 3: Figure S3). Fluorescence of both IRFP-expressing bacterial species in the separate mice was detected and differentiated by spectral unmixing 5 min after administration (Fig. 9a). After 2 h, the mouse previously administered with IRFP682-expressing bacteria was further administered with 5.0 × 10^10^ cells of IRFP713-expressing bacteria. Signals of both bacterial species were detected and separated in a single mouse (Fig. 9b; middle mouse). In a separate experiment, the two bacterial species could also be differentiated in the intestine of the mouse that was administered with both bacterial species. Immediately following the administration of IRFP713-expressing bacteria, they were detected only in the stomach and at the beginning of the small intestine. IRFP682-expressing bacteria that were administered 2 h earlier, on the other hand, had already reached the caecum and large intestine, but were also still present in the stomach (Fig. 9c).

## Discussion

IRFP713 has been expressed in three bacterial hosts, two LAB (*L. lactis*, *Lb. plantarum*) and model commensal/pathogenic bacterium *E. coli*, via inducible or constitutive expression system. The identity of IRFP713 was confirmed by determining the spectral properties; these corresponded to those determined previously [8]. IRFP713 was codon-optimized for *L. lactis* and therefore optimally suited for expression in this organism, as confirmed by the highest normalized fluorescence intensity. The fluorescence intensity of the IRFP713-expressing cell culture correlated with the optical density (cell concentration), and can be used for bacterial quantification [27, 28]. A linear relationship between the fluorescence intensity and the cell concentration was observed in cultures of all three bacterial species over a broad range of bacterial concentrations, facilitating precise quantification of bacteria in the culture. The background fluorescence of control bacteria was very low and did not differ from that of the growth medium. The fluorescence intensity of the IRFP713-expressing *L. lactis* culture and viability of the bacteria were shown to persist for much longer (50 and 14 days, respectively) than the period of the animal experiments (maximum 24 h). This allows the assumption that the gradual decrease in fluorescence in animal experiments (described below) is due to bacterial secretion and not to IRFP713 degradation or bacterial cell lysis. The fluorescence is not necessarily related to viable bacteria [27]; however free IRFPs would probably be degraded or denatured under intestinal conditions. Intestinal bacterial viability was confirmed by successful isolation of the bacteria from different parts of the mouse intestine.

IRFP713 enables excitation/emission in the near-infrared region (690/713 nm), which should minimize the autofluorescence of background tissue. In practice, however, the autofluorescence in this spectral range was still considerable and prevented exact localization of the signal source and estimation of its strength. This was partly circumvented by the use of a chlorophyll-minimized (alfalfa-free) diet [29]. The fluorescence intensity could be further increased by using nude or shaved mice; however, this was not considered in the present research. The IRFP713 signal was readily distinguished from the background by using spectral unmixing in reflectance (epifluorescence) imaging.

Different imaging modalities were employed to monitor the fate of the bacteria after administration to mice. Epifluorescence imaging is straightforward and fast. However, quantification of the fluorescence signal is limited, as it depends on both the fluorescence intensity of the source and its depth inside the animal body (distance from the surface) [4]. While the ratio of fluorescence of IRFP713-expressing *L. lactis* and *Lb. plantarum* measured in vivo corresponded to that determined in vitro, the fluorescence intensity of IRFP713-expressing *E. coli* was lower. Epifluorescence imaging of three mice that were administered simultaneously with IRFP713-expressing *L. lactis* resulted in considerable standard errors. Nevertheless, the time dependent decrease in fluorescent signal caused by bacterial secretion was in agreement with other approaches that were used (ex vivo imaging of intestines, fluorescence tomography). Epifluorescence imaging was used primarily for qualitative localization of the signal source, rather than source quantification. When comparing epifluorescence images of intact animals with those of mice with open abdominal cavities, or with tomographic images, considerable agreement was observed. However, despite effective separation of IRFP713 fluorescence from the tissue autofluorescence, reconstruction of the exact position of the source of the signal and its annotation to a specific organ is difficult. During the first hour after administration of different bacterial species, when the bacteria traverse the small intestine (discussed below) the movements of the bacterial mass could be observed, but reconstitution of its positioning in the small intestine was limited. Annotation of the source of the signal to a specific organ could be improved by the use of computed tomography scan.

Exact positioning of all three species of fluorescent bacteria was achieved by epifluorescence ex vivo imaging of isolated intestines, in which the tissue thickness is minimal. The quantification achieved by measuring radiant efficiencies in different parts of the intestine was verified by isolating and determining the count of viable bacteria in the corresponding intestinal parts. Good correlations were observed for caecum and large intestine, and somewhat lower correlations for stomach and small intestine, probably due to faster transit through the latter two organs. However, ex vivo imaging is an approach that only partially fulfils the aim of the present study, namely in vivo imaging.

To resolve the exact positioning of the bacteria in the whole animal fluorescence tomography (FLIT), that requires transillumination fluorescence measurements, was applied. FLIT enabled spatial reconstruction of IRFP713-expressing bacteria at given time points. Due to the complexity, spectral unmixing combined with FLIT is not feasible in time-course experiments and was not employed. Nevertheless, comparison of FLIT with epifluorescence images of whole animals and animals with an open abdominal cavity revealed very good agreement between different imaging techniques. This suggests that the FLIT technique is suitable for in vivo monitoring of IRFP-expressing bacteria.

All imaging modalities were employed for determining the time-course of transit of three bacterial species in live mice in a 24 h time window. The results of epifluorescence imaging of live mice, tomographic (FLIT) imaging, ex vivo epifluorescence imaging of isolated intestines and culturing of the bacteria from different parts of the intestine were in agreement. To summarize, bacterial mass reached the stomach immediately after administration. After approximately 1 h, bacteria passed through the small intestine and reached the caecum. In some experiments longer bacterial retention in the stomach was observed, possibly due to inter-animal differences in feeding prior to administration, or to coprophagy. From the caecum the bacteria entered the large intestine and were gradually secreted from the organism. Bacteria were barely detectable 6–8 h after the administration, using imaging techniques, due to considerable secretion and dilution, and were no longer detected after 24 h. The observed intestinal transit data correspond to those reported [29]. No major differences were observed between different bacterial species, as inferred from the ex vivo epifluorescence imaging of isolated intestines. However, the differences in normalized fluorescence intensity between bacterial species somewhat hindered comparison of transit times. This was particularly evident with *E. coli* that had the lowest normalized fluorescence and therefore resulted in the apparently fastest clearance from the organism.

One of the potential advantages of in vivo fluorescence imaging is the concomitant monitoring of multiple fluorescent proteins with different spectral properties. A palette of different IRFPs has recently been introduced [9]. The gene for the IRFP variant IRFP682 was obtained by site-directed mutagenesis and expressed in *L. lactis*. Its identity was confirmed by spectral properties in the bacterial culture that corresponded to those reported previously [8]. The considerable spectral overlap of IRFP682 and IRFP713 prevents distinguishing them with FLIT. On the other hand, the epifluorescence signals of IRFP682 and IRFP713 can be distinguished from each other by spectral unmixing. A population of *L. lactis* expressing IRFP713 was distinguished from one expressing IRFP682 in vivo in mice and ex vivo in isolated intestine. However, it should be noted that the two bacterial species can be more effectively resolved by spectral unmixing if they are sufficiently separated spatially. The fluorescence signal is less reliably unmixed if there is a significant bacterial overlap (e.g., bacterial mixture in a single organ). The resolution of bacterial populations could be further improved by using photoacoustic tomography [30].

## Conclusions

The feasibility of using fluorescence for in vivo monitoring of LAB in mice has been demonstrated in the present work. Near-infrared fluorescent protein IRFP713 was expressed in two LAB species (*L. lactis*, *Lb. plantarum*) and in a model commensal/pathogen bacterium, *E. coli*, and was detected directly in a bacterial culture without the need for removal of the growth medium, indicating its potential as a reporter protein in various bacteria. All three bacterial species were imaged in live mice with IVIS Spectrum, using fluorescence reflectance imaging (epifluorescence) or fluorescence tomography. The data were supplemented by epifluorescence imaging of isolated intestines ex vivo. The ability to monitor the gastrointestinal transit time of bacteria was demonstrated, as well as their spatial localization. Viable bacteria were isolated and cultured from different parts of the intestine and their quantity corresponded to the fluorescence signal determined by imaging. Two IRFPs, differing in their spectral properties, were expressed in *L. lactis*. Populations of bacteria expressing IRFP713 and IRFP682 were distinguished in vivo, enabling their concomitant monitoring. A platform for future studies of probiotic and theranostic effects of LAB in mouse disease models, as well as their interaction with commensal or pathogenic bacteria, has thus been established.

## Methods

### Bacterial strains, media and culture conditions

The bacterial strains used in this study are listed in Table 1. *L. lactis* NZ9000 was grown at 30 °C in M-17 medium (Merck) supplemented with 0.5 % glucose (GM-17) and 10 µg/mL chloramphenicol without aeration. *Lactobacillus plantarum* ATCC 8014 was grown at 37 °C in De Man, Rogosa and Sharpe (MRS) medium (Merck) supplemented with 10 µg/mL chloramphenicol, without aeration. *E. coli* DH5α was grown at 37 °C with aeration in lysogeny broth (LB) supplemented with 100 µg/mL ampicillin. Media were supplemented with 15.5 µg/mL biliverdin HCl (Sigma Aldrich).

**Table 1:** Strains, plasmids, gene and primers

Bacteria, plasmid, or gene	Relevant features or sequence (5′–3′)	References or sources
Bacteria		
*E. coli* DH5α	endA1 glnV44 thi-1 recA1 relA1 gyrA96 deoR F- Φ80d*lacZ*ΔM15 Δ(*lacZYA*-*argF*)U169, hsdR17(rK- mK +), λ–	Invitrogen
*L. lactis* NZ9000	MG1363 *nisRK* Δ*pepN*	NIZO [35, 36]
*Lb. plantarum* ATCC 8014	Wild type	ATCC
Plasmid		
pNZ8148	pSH71 derivative, P*nisA,* CmR, nisin-controlled expression	NIZO [35, 36]
pNZRK	pNZ8148 containing *nisR* and *nisK* genes	This work
pNZ-IRFP713	pNZ8148 containing *irfp713* gene	This work
pNZ-IRFP682	pNZ8148 containing *irfp682* gene	This work
pNZRK-IRFP713	pNZ8148 containing *irfp713, nisR* and *nisK* genes	This work
pMA-T-IRFP713	pMA-T containing *irfp713* gene	This work
pMSP3545	Em^r^, P_*nisA*_, *nisRK*, *Nco*I for translational fusion, ColE1 and pAMβ1 replicons	[37]
pMSP3545-IRFP713	pMSP3545 containing *irfp713* gene	This work
pGEM::CP25-IRFP	pGEM-T Easy containing *irfp713* gene under the control of CP25 promoter	[27]
Gene		
*irfp713*	CCATGGCTGAGGGATCTGTAGCTCGTCAACCTGATTTACTTACTTGTGACGATGAACCTATTCATATTCCAGGTGCTATTCAACCACACGGACTTTTATTAGCTCTTGCCGCTGACATGACTATCGTCGCTGGATCAGATAATTTACCTGAATTGACTGGTTTAGCAATTGGAGCCCTTATTGGACGATCAGCAGCAGATGTTTTTGATTCAGAAACTCATAATCGTCTTACTATTGCATTGGCAGAACCTGGTGCTGCAGTAGGTGCTCCTATTACAGTAGGTTTCACTATGCGTAAGGATGCTGGTTTTATTGGTTCTTGGCATCGTCATGATCAACTTATTTTTTTAGAGTTGGAACCACCACAAAGAGACGTTGCAGAGCCTCAAGCTTTTTTTCGTCGTACTAATTCAGCAATTCGTAGACTTCAAGCTGCTGAAACTCTTGAATCTGCATGTGCAGCAGCAGCACAAGAGGTACGAAAAATTACAGGTTTTGATAGAGTTATGATTTACAGATTTGCCTCAGACTTTTCTGGTGAAGTAATCGCAGAGGATAGATGTGCCGAGGTTGAAAGTAAATTGGGATTGCATTATCCTGCCAGTACTGTTCCAGCCCAAGCACGTCGTCTTTATACTATTAATCCTGTTAGAATTATCCCAGATATTAATTATCGACCAGTTCCAGTTACTCCTGACTTAAATCCTGTAACTGGTAGACCTATTGACTTGTCATTTGCCATCTTACGATCTGTTTCACCTGTTCATTTAGAGTTTATGCGTAATATTGGTATGCATGGTACTATGTCTATCTCAATCCTTCGAGGAGAACGTTTATGGGGACTTATTGTTTGTCATCATAGAACACCTTATTATGTCGATTTAGATGGACGTCAAGCTTGTGAATTAGTTGCTCAAGTCTTGGCTTGGCAAATTGGTGTAATGGAAGAATAATCTAGA	This work
Primers		
nisR-F	GATGATAAGCTGTCCAAAC	This work
nisK-R	TTTAGGATAACTTCTGCCC	This work
682-1F	CTCAGACTTTTCTGGTGTAGTAATCGCAGAGGATAG	This work
682-2F	GCATTATCCTGCCAGTGCTGTTCCAGCCCAAGC	This work
682-3F	CCATCTTACGATCTGTTTCACCTTGTCATTTAGAGTTTATGCG	This work
682-1R	CTATCCTCTGCGATTACTACACCAGAAAAGTCTGAG	This work
682-2R	GCTTGGGCTGGAACAGCACTGGCAGGATAATGC	This work
682-3R	CGCATAAACTCTAAATGACAAGGTGAAACAGATCGTAAGATGG	This work

### Bacterial viability

The number of colony forming units (CFU)/cm^2^ was determined by using the drop-plate method [31]. Long term stationary culture viability was determined by plating tenfold dilutions without antibiotic. To determine CFU/cm^2^ in different parts of the intestine the contents of stomach, small intestine, caecum and large intestine were aseptically squeezed out and resuspended in 500 µL PBS, vortexed vigorously and briefly centrifuged (4 s spin). Ten-fold dilutions of the supernatant were plated on chloramphenicol-containing GM-17 plates using the drop-plate method [31]. The CFU/cm^2^ was normalized to the weight of the intestinal content.

### IRFP cloning

The IRFP amino acid sequence [8] was back-translated and codon-optimized for *L. lactis*, yielding *irfp713* gene, which was obtained from Geneart (Table 1). The gene was cloned to pNZ8148, pMSP3545 and pNZRK via *Nco*I/*Xba*I sites. pNZRK was prepared by digesting pNZ8148 with *Sal*I, blunting and blunt-end ligation with *nisRK* PCR amplicon. The latter was obtained by using nisR-F/nisK-R primer pair on pMSP3545 template. pNZRK was prepared for the use of PnisA promoter in *Lb. plantarum* ATCC 8014, because its genome does not contain *nisRK* genes. pMA-T-IRFP713 was used as a template to introduce mutations E180V, T202A and V254C by using QuikChange II Site-Directed Mutagenesis Kit (Stratagene). Primer pairs 682-1F/682-1R, 682-2F/682-2R and 682-3F/682-3R were used sequentially to obtain *irfp682* gene [8]. All plasmids were verified by nucleotide sequencing performed by GATC Biotech (Germany). KOD polymerase was used for PCR amplification. Restriction enzymes were from New England Biolabs and Fermentas. *L. lactis* and *Lb. plantarum* were transformed according to Holo [32] and Berthier [33], respectively, with electroporation, using Gene Pulser II apparatus (Biorad). *E. coli* was transformed with heat-shock.

### Expression of IRFP variants

Overnight cultures of bacteria harboring appropriate plasmids were diluted (1:100) in fresh medium and grown to optical density (OD_600_) 2.50–3.00. Required exogenous chromophore biliverdin was provided in bacterial growth medium when culturing bacteria for both in vitro and in vivo experiments. *L. lactis* cultures were induced with 25 ng/mL nisin (Fluka) at OD_600_ = 0.80 and incubated for a further 3 h; no induction was necessary for *Lactobacillus* and *Escherichia* cultures. Bacterial cultures were centrifuged at 5000*g*, resuspended in an appropriate volume of 10 % w/v sucrose solution and stored at 4 °C before administration.

### Measurement of IRFP fluorescence in bacterial cultures

Aliquots of cell cultures (200 µl) were transferred to black, flat-bottom 96-well plates (Greiner). Fluorescence was measured on an Infinite M1000 microplate reader (Tecan) [27], with excitation/emission at 690/713 nm for IRFP713, or 663/682 nm for IRFP682. Fluorescence intensity was normalized to a cell density OD_600_ = 1.0. Excitation and emission spectra were recorded between 600 and 800 nm at 2 nm intervals, using the appropriate excitation and emission maxima (663/682 nm for IRFP682; 690/713 nm for IRFP713). All the measurements were made in triplicate.

### Imaging of IRFP-expressing bacteria in vivo in mice

Fifty 8 week-old FVB mice were bred in the animal facility at the Jozef Stefan Institute. They were housed in pathogen-free conditions, with food and water ad libitum. Alfalfa-free Teklad global rodent diet 2016 was used to minimize background intestinal fluorescence [34] at least 4 days before the start of experiments. Maximum volumes of 200 μL of bacteria were administered by oral gavage. Bacteria were kept at 4 °C for 2 days prior to administration to ensure stable fluorescence.

An IVIS Spectrum in vivo imaging system (PerkinElmer) was used for fluorescence imaging of mice. Mice were anesthetized with isoflurane (Forane). Spectral unmixing of IRFP713 and background fluorescence signals was performed by recording sequences of images using the following excitation/emission filter pairs: 675/720 nm, 675/740 nm, 675/760 nm, 605/660 nm, 605/680 nm, 605/700 nm, 605/720 nm, 605/740 nm and 605/760 nm. Spectral unmixing of IRFP682, IRFP713 and background fluorescence signals was performed by recording a sequence of images using the following excitation/emission filter pairs: 640/680 nm, 640/700 nm, 640/720 nm, 640/740 nm, 640/760 nm, 640/780 nm, 675/720 nm, 675/740 nm, 675/760 nm, 605/660 nm, 605/680 nm, 605/700 nm, 605/720 nm, 605/740 nm and 605/760 nm. Instrument background fluorescence was removed by using the adaptive fluorescence background subtraction tool. Exposure time was adjusted to obtain count numbers between 600 and 60,000. The region of interest (ROI) was set manually and radiant efficiency (photons s^−1^ cm^−2^ steradian^−1^ per μW cm^−2^) was determined when appropriate. Fluorescence tomography (FLIT) was performed by recording a trans-illumination sequence of eight images. Images were analyzed using Living Image, version 4.3.1.

When required, mice were euthanized by cervical dislocation. This was followed by exposure of the abdominal cavity and removal of the intestine from stomach to rectum.

### Ethics statement

All experimental procedures were carried out in accordance with institutional and national guidelines and were approved by the Administration of the Republic of Slovenia for food safety, veterinary sector and plant production (Permit No. U34401-2/2014/6). All the efforts were made to minimize animal suffering.

## Additional files

10.1186/s12934-015-0376-4 Time-dependent viability (A) and stability of IRFP713 fluorescence (B) of the stationary phase *L. lactis* culture expressing IRFP713 (red), or empty vehicle control (without *irfp713* gene; blue). The bacterial culture was stored at 4°C for 56 days.
10.1186/s12934-015-0376-4 Representative example of spectral unmixing of IRFP713 signal from background signal. A: collection of images recorded with different filter pair combinations (see Methods). B: unmixed background (left) and IRFP713 signal (right). Color bar indicates radiant efficiency.
10.1186/s12934-015-0376-4 Representative example of spectral unmixing of IRFP713, IRFP682 and background signal. A: A collection of images recorded with different filter pair combinations (see Methods). B: unmixed background (top), IRFP713 (bottom left) and IRFP682 signal (bottom right). Color bar indicates radiant efficiency.

## Abbreviations

BV: biliverdin
CFU: colony-forming unit
COMP: composite image
ECO: *Escherichia coli*
FLIT: fluorescence imaging tomography
GFP: green fluorescent protein
GM17: M17 medium with 0.5 % glucose
IRFP: infrared fluorescent protein
LAB: lactic acid bacteria
LAC: *Lactococcus lactis*
LB: lysogeny broth
MRS: De Man, Rogosa and Sharpe medium
NIS: nisin
OD_600_: optical density at 600 nm
PLA: *Lactobacillus plantarum*
