# Supplementary figures and images for: In vivo imaging of Lactococcus lactis, Lactobacillus plantarum and Escherichia coli expressing infrared fluorescent protein in mice
Source: Microb Cell Fact. 2015 Nov 14;14:181. doi: 10.1186/s12934-015-0376-4 (PMC4650289; doi:10.1186/s12934-015-0376-4)

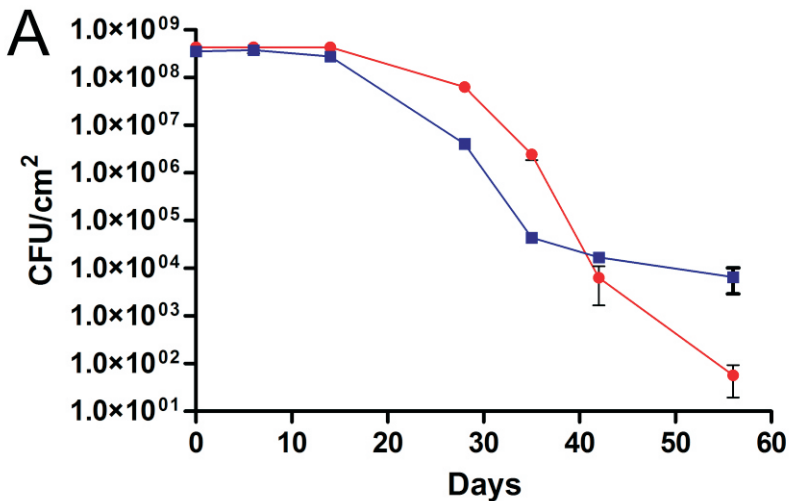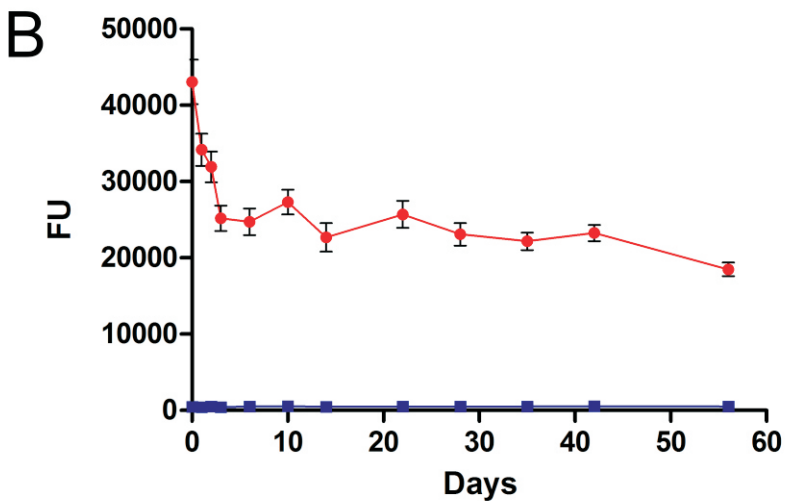

Supplement: Supplementary file 1 — 10.1186/s12934-015-0376-4 Time-dependent viability (A) and stability of IRFP713 fluorescence (B) of the stationary phase L. lactis culture expressing IRFP713 (red), or empty vehicle control (without irfp713 gene; blue). The bacterial culture was stored at 4°C for 56 days. [file 12934_2015_376_MOESM1_ESM.pdf]
