# Supplementary material for: In vivo imaging of Lactococcus lactis, Lactobacillus plantarum and Escherichia coli expressing infrared fluorescent protein in mice
Source: Microb Cell Fact. 2015 Nov 14;14:181. doi: 10.1186/s12934-015-0376-4 (PMC4650289; doi:10.1186/s12934-015-0376-4)
Supplement: Supplementary file 2 — 10.1186/s12934-015-0376-4 Representative example of spectral unmixing of IRFP713 signal from background signal. A: collection of images recorded with different filter pair combinations (see Methods). B: unmixed background (left) and IRFP713 signal (right). Color bar indicates radiant efficiency. [file 12934_2015_376_MOESM2_ESM.pdf]

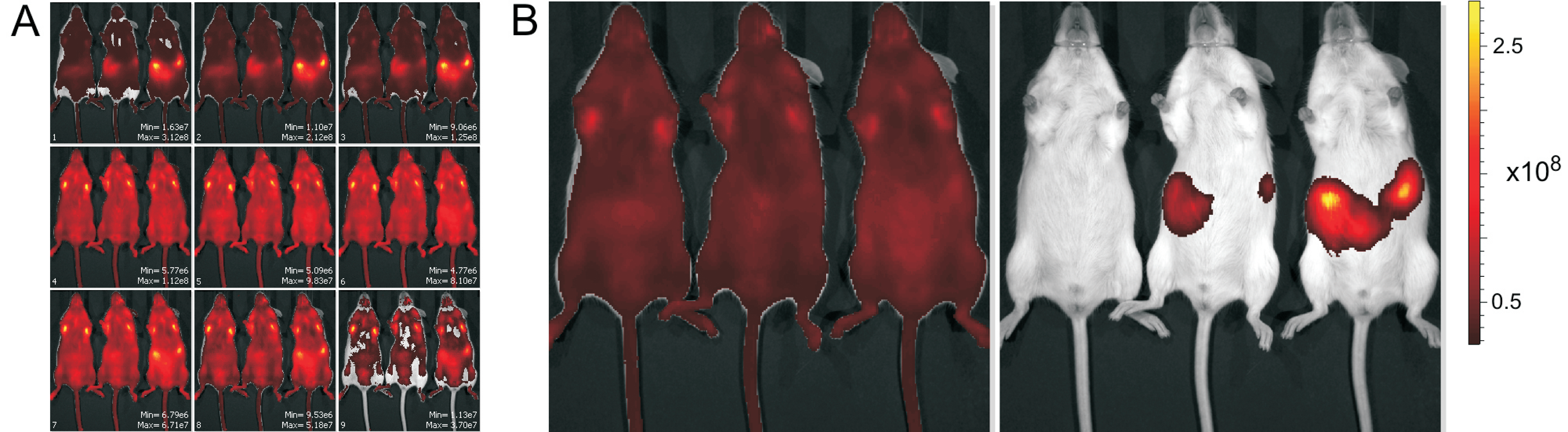
A
B
1
Min= 1.63e7
Max= 3.12e8
2
Min= 1.10e7
Max= 2.12e8
3
Min= 9.06e6
Max= 1.25e8
4
Min= 5.77e6
Max= 1.12e8
5
Min= 5.09e6
Max= 9.83e7
6
Min= 4.77e6
Max= 8.10e7
7
Min= 6.79e6
Max= 6.71e7
8
Min= 9.53e6
Max= 5.18e7
9
Min= 1.13e7
Max= 3.70e7
2.5
x10^8
0.5
