# Supplementary material for: In vivo imaging of Lactococcus lactis, Lactobacillus plantarum and Escherichia coli expressing infrared fluorescent protein in mice
Source: Microb Cell Fact. 2015 Nov 14;14:181. doi: 10.1186/s12934-015-0376-4 (PMC4650289; doi:10.1186/s12934-015-0376-4)
Supplement: Supplementary file 3 — 10.1186/s12934-015-0376-4 Representative example of spectral unmixing of IRFP713, IRFP682 and background signal. A: A collection of images recorded with different filter pair combinations (see Methods). B: unmixed background (top), IRFP713 (bottom left) and IRFP682 signal (bottom right). Color bar indicates radiant efficiency. [file 12934_2015_376_MOESM3_ESM.pdf]

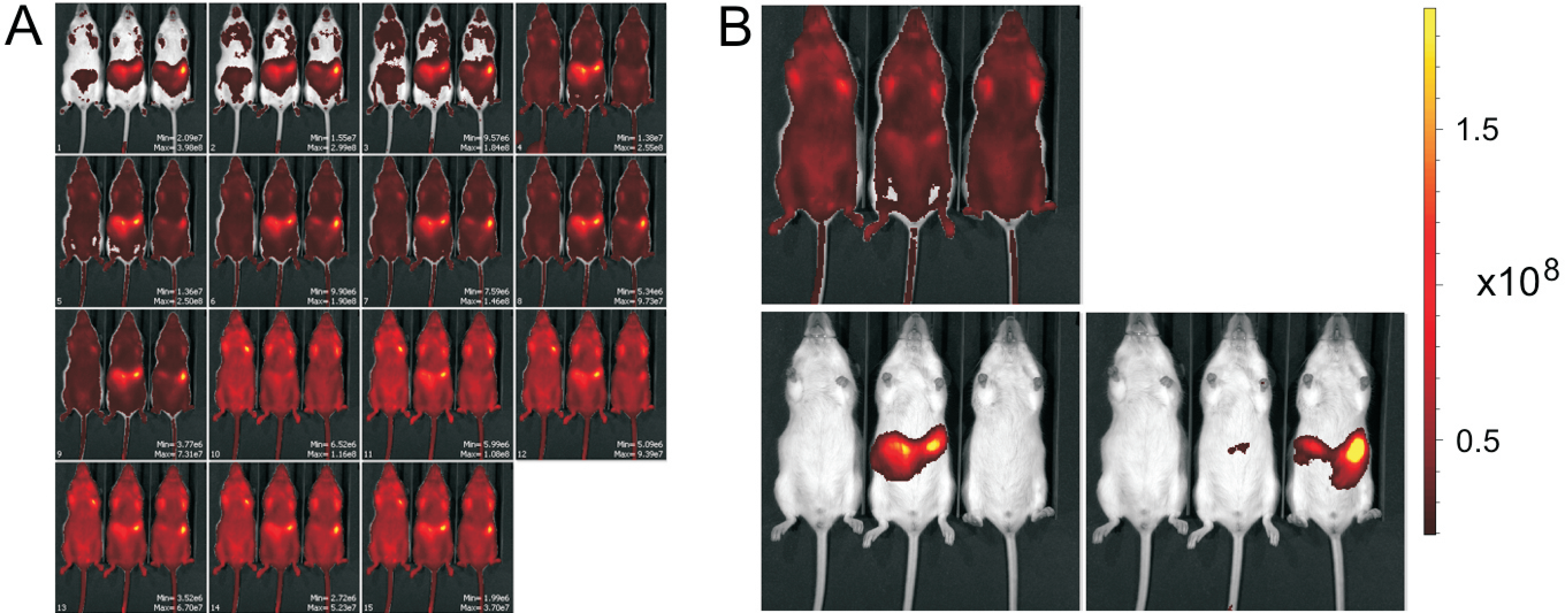
A
1
Min= 2.09e7
Max= 3.98e8
2
Min= 1.55e7
Max= 2.99e8
3
Min= 9.57e6
Max= 1.84e8
4
Min= 1.38e7
Max= 2.55e8
5
Min= 1.36e7
Max= 2.50e8
6
Min= 9.90e6
Max= 1.90e8
7
Min= 7.59e6
Max= 1.46e8
8
Min= 5.34e6
Max= 9.73e7
9
Min= 3.77e6
Max= 7.31e7
10
Min= 6.52e6
Max= 1.16e8
11
Min= 5.99e6
Max= 1.08e8
12
Min= 5.09e6
Max= 9.39e7
13
Min= 3.52e6
Max= 6.70e7
14
Min= 2.72e6
Max= 5.23e7
15
Min= 1.99e6
Max= 3.70e7
B
1.5
x10^8
0.5
